# Tin contamination in sediments of Lake Zurich: source, spread, history and risk assessment

**DOI:** 10.1186/s00015-024-00471-6

**Published:** 2024-12-24

**Authors:** Remo L. Roethlin, Aurélia C. E. Meister, Adrian Gilli, Sinikka T. Lennartz, Helen Eri Amsler, Maria Dittrich, Bernhard Wehrli, Maria Schönbächler, Nathalie Dubois

**Affiliations:** 1 Department of Surface Waters Research and Management, Eawag, Überlandstrasse 133, Dübendorf, 8600 Switzerland; 2 Geological Institute, Department of Earth and Planetary Sciences, ETH Zurich, Sonneggstrasse 5, Zurich, 8092 Switzerland; 3 Institute of Geochemistry and Petrology, Department of Earth and Planetary Sciences, ETH Zurich, Sonneggstrasse 5, Zurich, 8092 Switzerland; 4 Department of Earth and Planetary Sciences, ETH Zurich, Sonneggstrasse 5, Zurich, 8092 Switzerland; 5 Institute for Chemistry and Biology of the Marine Environment, University of Oldenburg, Carl-von-Ossietzky-Straße 9-11, Oldenburg, 26129 Germany; 6 University Library, University of Bern, Hochschulstrasse 6, Bern, 3012 Switzerland; 7 Biogeochemistry Group, Department of Physical & Environmental Sciences, University of Toronto Scarborough, 1065 Military Trail, Toronto, M1C 1A4 Canada; 8 Department of Surface Waters Research and Management, Eawag, Seestrasse 79, Kastanienbaum, 6047 Switzerland; 9 Department of Environmental Systems Science, ETH Zurich, Universitätstrasse 16, Zurich, 8092 Switzerland

**Keywords:** Tin, Sn, Contamination, Lake sediments, Lake Zurich, Silk industry

## Abstract

Industrial activities of a silk dyeing factory in Thalwil, on the shore of Lake Zurich, Switzerland, caused extreme Sn contamination of lake sediments. In this study, we determine the contamination source, spread, and age using a multiproxy approach. We used X-ray fluorescence spectroscopy (XRF) core scanning and further geochemical analyses to assess the contamination spreading and thickness in the sedimentary column. We found elevated Sn levels throughout sediments of Lake Zurich, ranging from 177 $\hbox {g} \,\hbox {kg}^{-1}$ in front of the former silk factory to 0.05 $\hbox {g} \,\hbox {kg}^{-1}$ at the southeast end (background: ca. 0.006 $\hbox {g}\, \hbox {kg}^{-1}$). The rapid concentration drop away from the shore suggests quick precipitation of a sparingly soluble inorganic Sn compound, which is confirmed by Scanning Electron Microscope Imaging in tandem with Energy-dispersive XRF spectroscopy (SEM-EDX) data. The Sn XRF profile of a varved core indicates a contamination onset in the early 1890s, a maximum around 1900, and a gradual decrease to low levels in the 1940s. High Sn concentrations in turbidite layers from the deep basin indicate that mass movements physically remobilised Sn. However, in stable conditions, in-situ porewater measurements (conc. < 0.5 $\upmu \hbox {g}\, \hbox {L}^{-1}$) using dialyse plates show little Sn remobilisation into the lake water (0.05 $\hbox {mg} \,\hbox {a}^{-1} \,\hbox {m}^{-2}$). The low remobilisation, reducing conditions, and high sulphide contents in the contaminated layers suggest that Sn is firmly bound to the sediments. Combined with the low toxicity of Sn, we conclude that the Sn contamination poses no threat to lake biota or drinking water production.

**Supplementary Information:**

The online version contains supplementary material available at 10.1186/s00015-024-00471-6.

## Introduction

While heavy metals like Sn are naturally present in the geosphere, human exploitation has greatly increased the amount and concentration of those metals released into the environment, especially with the advent of the Industrial Revolution in the middle of the 19^th^ century (Smol, 2008). The increase of those toxic and persistent contaminants is of global concern and raises the need to understand, prevent, and potentially remediate such contaminations.

Inorganic Sn is naturally present in the Earth’s crust at low concentration, around 2.1 ${\hbox {mg}\, \hbox {kg}^{-1}}$ (Rudnick and Gao, 2014). Nonetheless, Sn was already mined to be incorporated in alloys like bronze or pewter since the late 4^th^ millennium BCE, at the beginning of the Bronze Age (Brügmann et al., 2017). Following the Industrial Revolution, the uses of Sn diversified. Over time, inorganic Sn had a wide range of industrial applications, from silk weighting (Hurst, 1892; Hacke, 2008) to solder or food can production (Cima, 2011). Unlike inorganic Sn, organic Sn compounds are toxic to various degrees (Winship, 1988; World Health Organization, 2020) and most are artificial. Since the synthesis of the first organotin in the middle of the 19^th^ century (Caseri, 2014), many compounds have been developed, like the extremely toxic tributyltin used in antifouling paints for ships from the 1960s to the early 2000s (Sousa et al., 2014). However, some organotins can be produced naturally though methylation of inorganic Sn by sulfate-reducing bacteria under anoxic conditions in the sediments (Gilmour et al., 1987).

Anthropogenic Sn can follow various pathways into the environment. In the case of tributyltin, Sn is freed by paint erosion directly in surface water. However, water bodies are also at risk of contamination through incineration or careless disposal of industrial waste, or by contact with contaminants transported in the atmosphere. Ultimately, Sn sinks to the sediments due to its low solubility and affinity to particulate matter (Sigg et al., 1987; Sousa et al., 2014). Therefore, vertical profiles in the sediments allow for the detailed reconstruction of the evolution and extent of the contamination, unless physical remobilisation occurs through mechanical mixing or bioturbation.

**Fig. 1:**
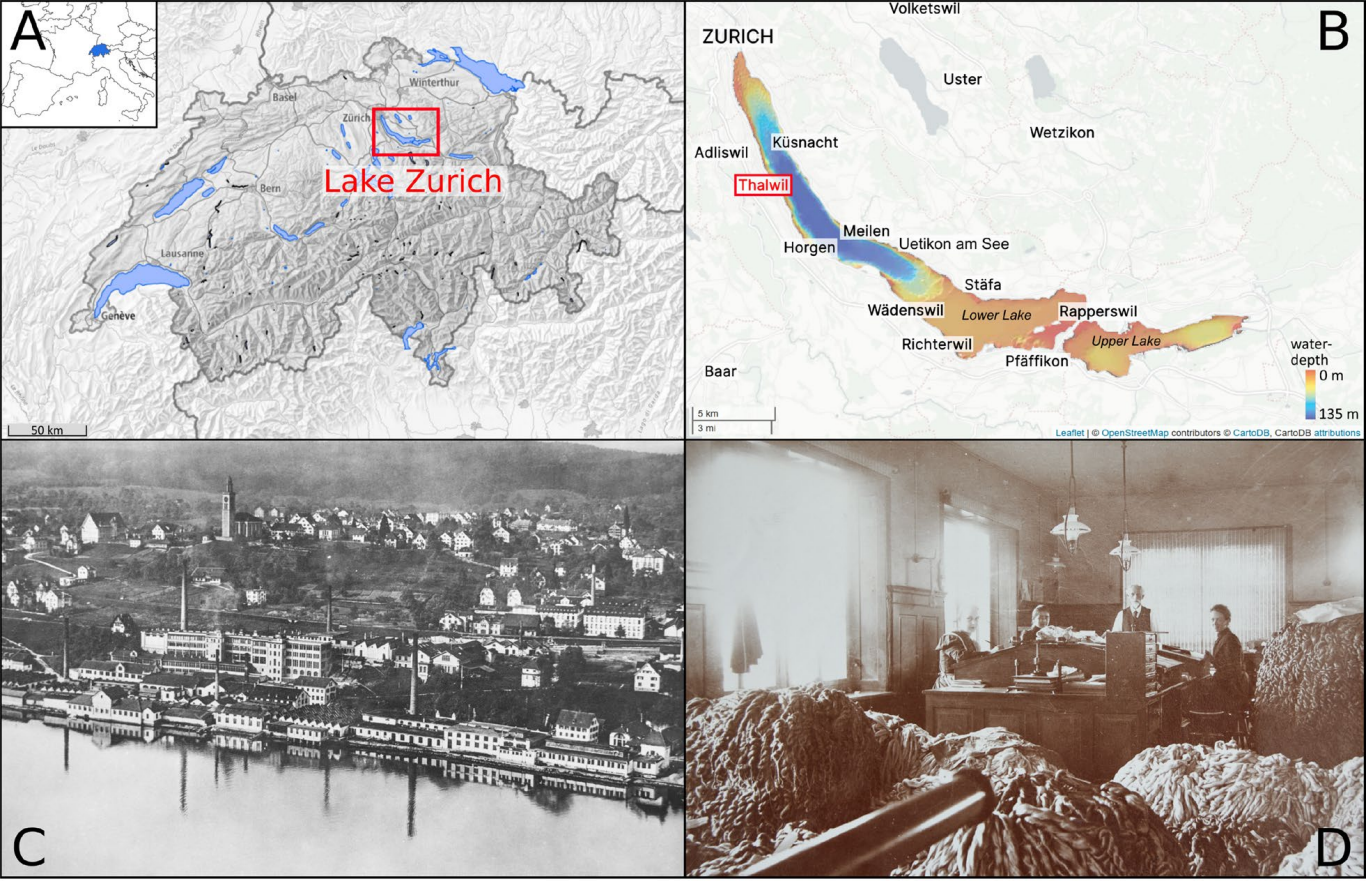
**A** Geographic location of Switzerland and Lake Zurich. **B** Location of Thalwil and bathymetry map of Lake Zurich. **C** Aerial view of the building complex of silk dyeing factory *Färberei Weidmann* in Thalwil around 1920. **D** Interior of the *Färberei Weidmann* around 1900 with the owner August Weidmann (second to the right). Map of Switzerland: Federal Office of Topography swisstopo. Bathymetry map modified after Strasser and Anselmetti (2008). Image courtesy **C** and **D**: Ortsmuseum Thalwil

In this study, we investigate a Sn contamination near Thalwil, Lake Zurich, Switzerland (Fig. 1A), where extreme Sn concentrations were discovered in the sediments during the remediation work of a tar contamination. Gasworks were responsible for the tar contamination (AWEL, 2019), but a tannery and a silk dyeing factory were also located near this location. Among those industries, the latter was known for requiring large quantities of Sn. Tin was also detected in contaminated sediments in front of Richterswil, in the southern part of Lake Zurich, along with several other heavy metals (Cr, Cu, Hg, Pb) (Roethlin et al., 2022a). However, the Sn concentration in the sediments near Thalwil appeared to be orders of magnitude higher than near Richterswil, thus warranting a separate study at this peculiar site.

Here, we synthesise existing knowledge on the Sn contamination near Thalwil and extend it with new results. In a first step, we investigate the history of the silk dyeing industry and its Sn use in Thalwil to allow a detailed comparison with the contaminated sediments. We then reconstruct the lateral spreading of the Sn contamination and the thickness of contaminated sediment layers using several sediment cores retrieved throughout Lake Zurich between 2005 and 2022. The presence of Sn in the distal cores retrieved from the deepest basin allows a relatively precise dating of the contamination based on varve chronology. The chronology of the Sn contamination in the sediment was then compared to the evolution of the silk industry in Thalwil.

We further investigate the depositional processes of the Sn by microscopically characterising the sediment particles, determining the grain size distribution, and analysing the bulk geochemical and trace metal composition of the sediment. Finally, we assess the risk posed by the Sn contamination in Lake Zurich, which is widely used for drinking water. In particular, we focus on its remobilisation potential by analysing the Sn concentration in pore water.

## Historical background

### Silk weighting and dyeing

Silk is produced by the larvae of silk moths, which spin their cocoon by secreting one single silk fibre that can reach 1 km in length (Hurst, 1892). Raw silk is composed of a shiny, proteinaceous fibre, the fibroin, that is surrounded by a sticky, often yellowish material, the sericin, also called gum. The gum is soluble in soapy water, allowing for reeling cocoons into skeins (see the foreground of the picture in Fig. 1D). During the 19^th^ and 20^th^ centuries, the golden age of the silk industry in Europe, the remaining gum was at least partially removed during the “degumming” or “boiling off” process: the skeins were first hung in hot soapy water (“stripping”), then in boiling soapy water (“boiling off”), and finally bleached if necessary (Hurst, 1892). The silk was then ready to be dyed.

However, removing the sericin during the degumming process resulted in a weight loss of up to 25% (Hacke, 2008). As the silk was sold by weight, this weight loss was often (over)compensated by treating the silk with artificial weighting compounds like Sn, in addition to the dyeing agents (Hacke, 2008). This practice was very popular, despite the risk of causing catastrophic damage to the fabric (Garside et al., 2010).

During the 19^th^ century, dyeing black on silk became the dominant branch of silk dyeing (Hurst, 1892), which also allowed for the highest weighting. Blacks were dyed using a combination of iron, logwood, and tannin baths. The tannins, obtained e.g. from chestnut or catechu, were mostly responsible for the weighting of the silk. Several tannin baths were alternated with iron baths to reach higher weights, but could only be used for dark shades (Hacke, 2008). The search for light or colourless weighting agents led to experiments with various substances like sugar or mineral salts, until the extremely successful tin weighting methods were discovered (Hacke, 2008).

There is no consensus in the literature on the date of the first use of tin weighting, but this method likely appeared in the 1870s (Hacke (2008) and references therein). Originally, tin was used for the dyeing of blue silk in the form of “tin crystals” (Hurst, 1892), that is, stannous chloride, SnCl_2_. While it could not be used as a weighting agent alone, it was observed to increase the effect of tannin weighting. For this, the silk was treated in a bath of tin crystals and tannins (e.g. catechu), followed by simple tannin baths. These tin-tannin solutions could only be used once and then had to be disposed of (Hurst, 1892). Combined with iron baths, the tin-tannin weighting could bring 1 kg raw silk to 11 kg finished black silk (Hacke, 2008).

The first colourless tin weighting method was introduced in 1883 and relied on the use of stannic chloride, SnCl_4_, followed by an alkaline rinse. Stannic chloride was sold in the form of ammonium chlorostannate (SnCl_4_ + 2 NH_4_Cl $\rightarrow$ (NH_4_)_2_SnCl_6_), the so-called “pink salt”, which gave the name to the pinking method. From the 1920s on, stannic chloride was mainly available as “butter of tin”, SnCl_4_ · 5 H_2_O, but the tin bath was still named pink bath (Hacke, 2008). The pinking method has the advantage of being applicable to raw or boiled-off silk that could then be bleached or dyed in any colour (Hacke, 2008).

H. J. Neuhaus further developed the pinking method into a new method, called “Neuhaus”, “dynamite”, or “tin-phosphate-silicate” weighting, patented in 1893 (Averell Harriman, 1948). Neuhaus introduced additional baths of sodium phosphate silicate (Hacke, 2008) or aluminium silicate (Garside et al., 2010), as well as a final silicate bath. White and coloured silks treated with the tin-phosphate-silicate method could be brought to weighting levels higher than ever without compromising the silk resistance (Hacke, 2008). This method rapidly became the most popular.

As the use of tin weighting methods grew, catastrophic damages to the fabric became more frequent. In particular in the years 1898–1902, the silk industry suffered from the rapid degradation of heavily tin weighted silks (Meister, 1905). As such, the weighting methods were not developed further, but the processes were optimised to reduce the waste of precious materials like Sn (Hacke, 2008). While a tin-tannin bath can only be used once, the Sn from other baths could be recuperated (Hurst, 1892). This recycling process proved to be particularly necessary during the supply shortage caused by the First World War (Schmid, 1959). In the course of the 20^th^ century, the use of weighting agents decreased due to improved regulation and control of weighted silks. In the early 1960s, synthetic polymer weighting began replacing tin weighting methods (Hacke, 2008). In addition, the global silk market shrunk following changes in fashion and the development of artificial silks. Nowadays, various methods are still applied to the silk, though the focus partially shifted from the weighting of the silk to the modification of fabric properties like handle or crease recovery (Hacke, 2008).

### Silk industry in Thalwil—*Färberei Weidmann*

In Thalwil (Fig. 1A and B), small dyeing factories can be traced back to the beginning of the 18^th^ century. However, the first significant silk dyeing industry was founded in 1867 by August Weidmann (1842-1928, Fig. 1D) and Jakob Julius Schwarzenbach (1844-1908) (Zwicky, 1995). Investing on technological progress and constant modernisation, they led their company *Schwarzenbach & Weidmann, Seidenfärberei* into becoming one of Europe’s most important silk centres.

The original factory building was significantly extended, even by reclaiming land from the lake by infilling (Schmid, 1959) (Fig. 1C). For rinsing the boiled silk, August Weidmann replaced the old method of dragging the silk behind a boat to directly rinse it in the lake, and instead installed pumps bringing the lake water into the factory (Schmid, 1959). In addition, August Weidmann recognised the importance of weighting in the silk preparation process and hired the ETH Zurich chemist Dr Otto Meister in the 1880s. During the next decade, several techniques were developed, improved, and patented. The success of the factory was growing over the next years, despite the withdrawal of Julius Schwarzenbach from the company—then renamed *Färberei Weidmann*—in 1895 (Schmid, 1959). Significant advancements in logwood black dyeing (tannin and tin-tannin weighting) allowed August Weidmann to win the “Grand Prix” at the *Exposition Universelle de Paris* in 1900 (“Exposants Suisses à l’Exposition Universelle de Paris 1900—liste des récompenses”, 1900). He was considered to be the best black dyer (Schmid, 1959), and Zurich was one of the largest silk centres in Europe, with Krefeld and Lyon (Hacke, 2008).

Difficult times began with the 20^th^ century, as complaints about weighted silks started rising. In addition, the resource shortage during the First World War forced the development of new techniques, notably to recuperate the Sn from the weighting baths (Schmid, 1959). A few years after August Weidmann’s passing in 1928, the *Färberei Weidmann* merged with other silk companies in 1933 to form the *Vereinigte Färbereien und Appretur AG Thalwil und Zürich* (Zwicky, 1995).

The silk dyeing industry in Thalwil had an important impact on the lake waters. Firstly, contaminants were directly released into the water by rinsing the silk in the wake of a boat. Secondly, the weighting and dyeing baths were likely emptied in the lake after use. Indeed, a resident of Thalwil, Adriana Berchtold, reported that she knows from still living persons that their skin would be coloured after a bath in the lake, over a wide zone around the shore of Thalwil. The dye would be so strong that they would know what colour was currently in use. A single shower would not be sufficient to wash out the colour (Adriana Berchtold, personal communication, 21 September 2021).

## Study site

Lake Zurich (406 m above sea level, 137 m maximum water depth, 68 ${\hbox {km}^{2}}$ surface area, 3.4 ${\hbox {km}^{3}}$ water volume) is a monomictic or dimictic peri-alpine lake in northeast Switzerland, with the City of Zurich and its outflow, the Limmat river, at its northwest end (Bossard et al., 2001). The lake basin is oriented from northwest to southeast, where the larger Lower Lake is separated from the Upper Lake (“Obersee”) by a moraine sill with a maximum water depth of 3 m. Upper Lake Zurich receives one significant inflow, the Linth channel, which connects Lake Zurich with Lake Walen. Upper Lake Zurich effectively acts as a settling basin for allochthonous material. This study focuses on a site in Thalwil at the western shore of the Lower Lake, approximately 10 km away from the city of Zurich. There, the lake quickly grows deeper and reaches a depth of 100 m in less than 400 m distance from the shore (Fig. 1B).

The water residence time of Lake Zurich is about 1.4 years (Wieland et al., 2001). Lake Zurich was originally oligotrophic. Varves appeared in the deep lake sediments in the 1890s as a result of the lake eutrophication following the development of the cities surrounding the lake (Boucherle and Züllig, 1983). Around 1965, the lake became mesotrophic due to the improved waste water treatments (Boucherle and Züllig, 1983). The sedimentation process is dominated by rapid particle coagulation resulting in high sedimentation rates (Weilenmann et al., 1989). Around 150 mass movements were observed in Lake Zurich over the last 17 000 years, revealing various processes affecting the area, such as earthquakes or floods (Strasser and Anselmetti, 2008). Several shore collapses or slumps occurred over the last 150 years as a result of human loading of sand or gravel on a weak lacustrine foundation, notably in Rüschlikon (1.5 km north of Thalwil), Oberrieden and Horgen (2.5 km and 4.5 km south of Thalwil, respectively) (Strasser and Anselmetti, 2008). The finer sediment mobilized during these mass movements and shore collapses usually goes into suspension and is deposited throughout the lake, forming distinct turbidite layers visible in the deep basin of Lake Zurich. Since Lake Zurich is the main drinking water supply for the City of Zurich, resuspension of shore sediments by mass movements poses a potential threat if these are contaminated.

## Materials and methods

This study aimed at analysing the Sn contamination near Thalwil and determining its source, extent, age, and remobilisation potential. The analyses were based on porewater measurements and sediment cores sampled in different campaigns between 2005 and 2022. Here, we summarised the different results gained over the years and extended existing knowledge with new results. To characterise the source and extent of the Sn contamination, we measured relative Sn concentration profiles in sediment cores using XRF corescanning. The absolute Sn concentration, as well as that of other heavy metals, was determined with geochemical analyses. The sediment particles were characterised using Scanning Electron Microscope Imaging in tandem with Energy-dispersive XRF spectroscopy (SEM-EDX). We determined the age of the contamination precisely with a varve chronology in a sediment core from the deep part of Lake Zurich. Finally, we evaluated the risks posed by the Sn contamination using *in-situ* porewater measurements coupled to grain size and bulk density measurements to estimate the potential of remobilisation.

### Coring and sample preparation

Sediment cores were taken in the years 2005 (core ZH05-57; used for scanning electron microscopy SEM and energy dispersive x-ray spectroscopy EDX), 2008–2010 (10 sediment cores; used for geochemical analyses and porewater diffusion measurements; see Supporting Information Figures A1 and A2), and during four coring campaigns between 2019 and 2022 (geochemical analyses, dating, XRF core scanning; see Supporting Information Figure A3). A complete list of used sediment cores with their respective coordinates is included in the Supporting Information (Table B1).

**Fig. 2:**
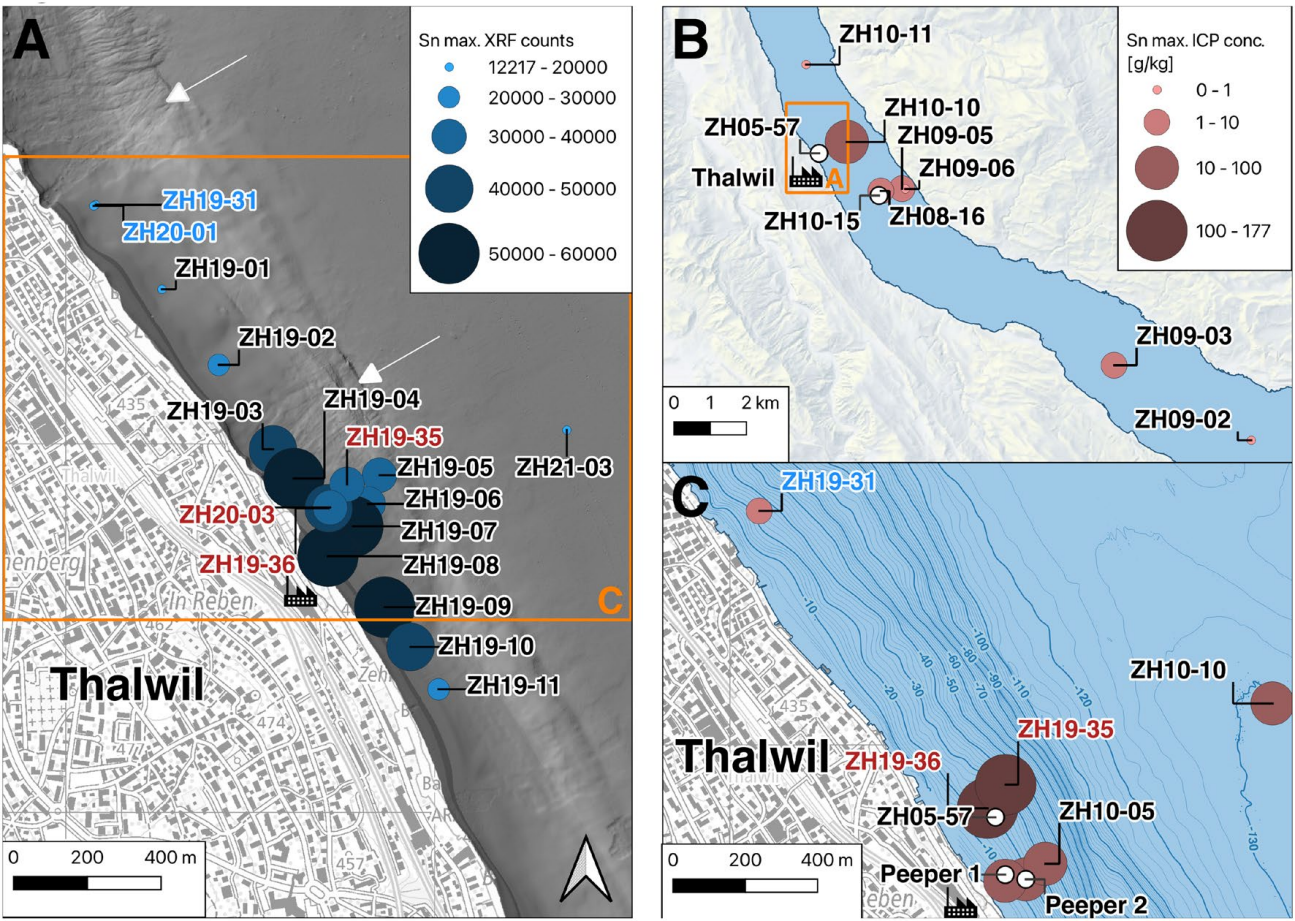
Overview of sediment cores and dialyse plate locations used in this study. **A** Maximum Sn XRF counts for selected cores close to the former silk factory in Thalwil. In red: contaminated core sites. In blue: uncontaminated core site (reference). In black: other sediment cores. White arrows: prominent underwater slides in the vicinity of the contamination, shown on a bathymetry map. Orange rectangle indicates location of panel **C**. **B** Overview of maximum ICP Sn concentrations in lower Lake Zurich. Orange rectanlge indicates location of panel **A**. **C** Detail of maximum ICP Sn concentrations close to the shore in Thalwil with the locations of the dialyse plates/peepers. The former silk factory is indicated with a factory icon. Maps: Federal Office of Topography swisstopo. Created with QGIS v3.22 (QGIS Development Team, 2016)

The coring locations were chosen around the known contamination in front of the former silk factory in Thalwil and throughout the lake to delimit the spreading of the Sn contamination (Fig. 2). Cores ZH19-35 and ZH19-36 were taken and sampled to represent the most contaminated area in the sediments in front of the factory (henceforth “contaminated site”), whereas ZH19-31 represents the less-contaminated area located further north along the shore (henceforth “uncontaminated site”). Sediment core ZH21-03 was taken in the deepest part (ca. 132 m) of Lake Zurich in front of Thalwil. The cores taken between 2008–2010 were used to estimate the spreading of the contamination throughout Lake Zurich. In particular cores ZH09-05 and ZH10-15 were retrieved from the central part of the deepest basin, about 2 km south east of Thalwil, and used in conjunction with ZH21-03 to determine the age of the contamination.

Cores were retrieved using a 62 mm gravity corer. GPS coordinates were recorded with a handheld device. Depth was estimated using the ship’s echo sounder and cross-checked with the publicly available bathymetry map of Lake Zurich (Strupler et al., 2018). The cores were opened longitudinally and kept in cold storage at ${4}\,{^\circ }\hbox {C}$. Core halves were photographed using a Jai CV L105 3CCD Colour Line Scan Camera in an Avaatech XRF core scanner, and core images were processed using Adobe Photoshop.

Cores ZH19-31, ZH19-35 and ZH19-36 were sub-sampled at 1 cm intervals for geochemical and grain size analysis; cores from earlier campaigns had been sampled in irregular intervals with respect to the maximum expected Sn concentrations after XRF scanning. After pea-sized fresh sediment samples were sub-sampled for grain size analysis, the remaining sediment material was frozen for 24 h, freeze-dried for 48 h in a Steris Lyovac GT2-E and ground using an agate mortar for the subsequent analyses.

### XRF core scanning

**Fig. 3:**
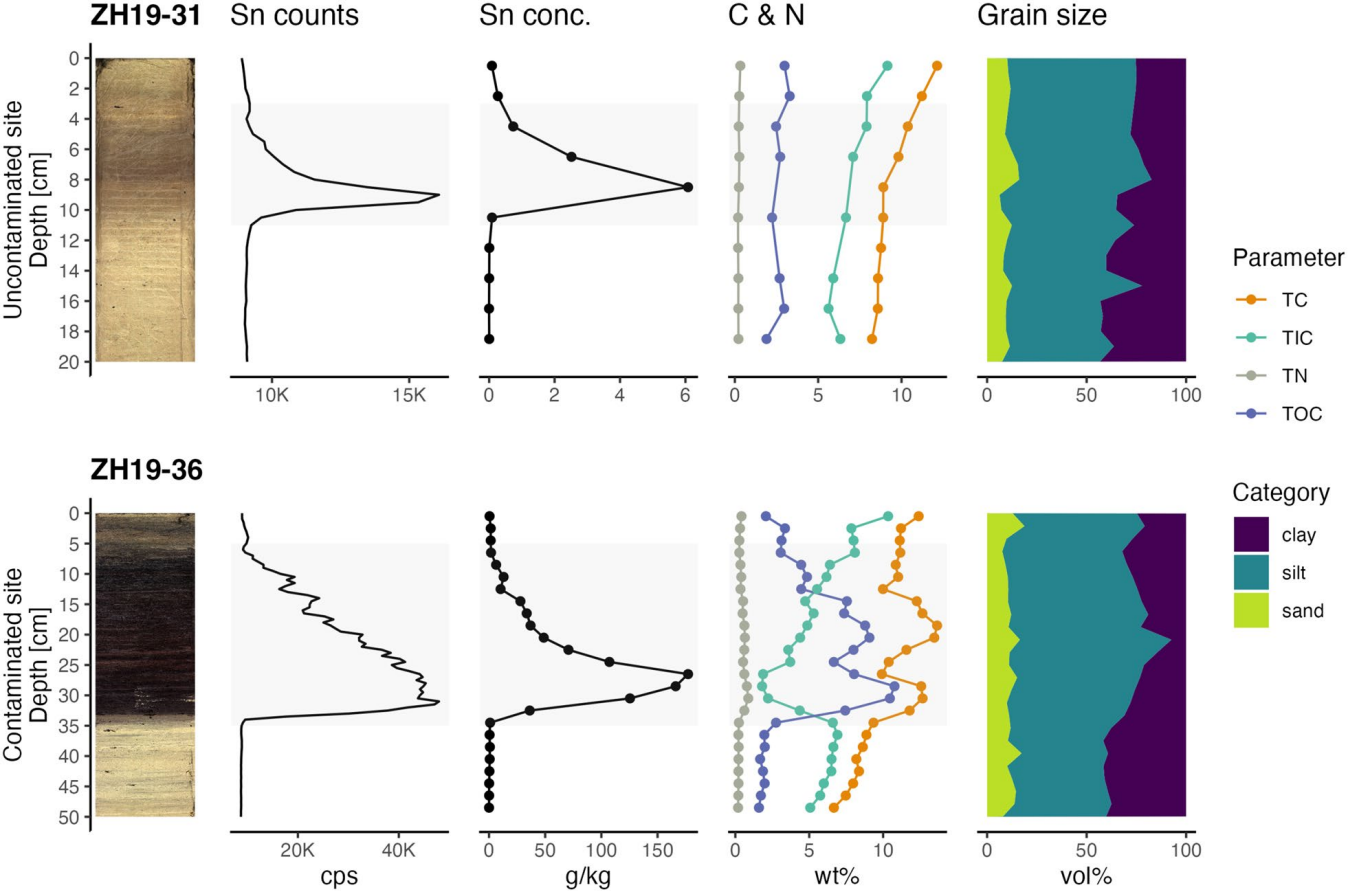
Overview of key data types for the uncontaminated site (ZH19-31) and from the heavily contaminated site (ZH19-36). From left to right: photograph of the sediment cores, Sn XRF counts, Sn concentrations, TC, TOC, TIC, TN, and grain size distribution profiles. The grey area indicates the period of Sn contamination in both cores

XRF core scanning was done using an Avaatech XRF core scanner (Richter et al., 2006; Roethlin et al., 2022a). A custom method was created (50 kV, 2000 mA, Sn-$K_\alpha$ line) to additionally measure Sn (Fig. 3). The spectral acquisition model for Sn was improved by using the bAxil software version 1.6.4. The spectral model analysis was implemented with a Gaussian peak shape function including escape peaks. Fitting chi square ($\chi ^2$) was set at 195 with iterations of 6. The fitting region includes the following region of interest (ROI) with boundaries: First channels (chn) = 1191, Last chn = 1316; First chn [keV] = 23.962, Last chn [keV]= 26.613. The down-core slit size was 1 cm (ZH19-1 - ZH19-11) and 5 mm (other cores), respectively. Due to the inhomogeneity of the sample matrix (all elements) and overlapping spectral peaks (Sn-$K_\alpha$), the counts for each element are not proportional to the counts of other elements within the same core or the counts of the same element in a different core (Hennekam et al., 2019; Weltje and Tjallingii, 2008). XRF file handling and statistical analyses were done with GNU R v4.2.0 (R Core Team, 2021), the tidyverse packages (Wickham et al., 2019) and the Avaatech XRF parser and interactive web app in the R package carrrotter (Roethlin et al., 2022b).

### Geochemical analyses

Discrete powdered samples from cores ZH19-31, ZH19-35 and ZH19-36 were extracted in Teflon tubes with a mixture of 0.5 mL H_2_O_2_ 30% and 2 mL HNO_3_ 65% using an ETHOS1 microwave and measured with a Spectro Arcos ICP-OES for multiple metals, including Cr, Cu, Pb, Sn, and Zn (Roethlin et al., 2022a). A multi-element standard was used for Cr, Cu, Pb, Zn, and a different standard for Sn. Samples from cores of the series ZH08-XX, ZH09-XX and ZH10-XX were digested in *aqua regia* (4 mL HNO_3_ 65% and 1 mL HCl 30%) using a Milestone mls 1200 mega microwave. The samples were measured using an Element 2 ICP-MS (Thermo Finnigan) and calibrated with a multi-element standard. Total carbon (TC) and total nitrogen (TN) were determined using an Elementar EA-IRMS, and total inorganic carbon (TIC) and total organic carbon (TOC) were determined using an Elementar soli TOC cube.

### Scanning electron microscope—energy dispersive X-ray spectroscopy (SEM-EDX)

We analysed the sediment samples in a field emission scanning electron microscope (Hitachi S 4500) equipped with an energy dispersive X-ray spectroscopy system (Oxford Instruments) for element analysis. Subsamples were dried, pestled and carefully dispersed on the cohesive surface of a support (diameter 10 mm). The subsamples were sputtered with carbon. We selected specific structures randomly and repeated microanalysis at least 10 times.

### Dating

**Fig. 4:**
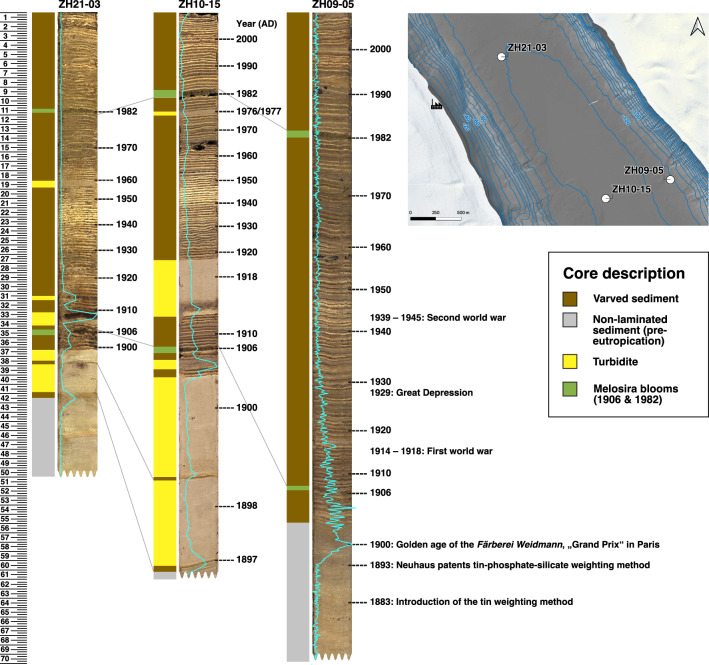
Age model for Sn contamination (the turquoise line represents scaled XRF scan profiles) based on varve counting for cores ZH21-03, ZH10-15 (Naeher et al., 2013) and ZH09-05. The map shows the locations, depths and structures of the underground (bathymetry map) for the three cores. Due to the slightly elevated location of ZH09-05, the stratigraphy is not interrupted by turbidites

Biogenic varves were counted in core ZH21-03 and ZH09-05, retrieved from the deepest part of Lake Zurich. Varves allow for a more precise dating in the distal cores than radiometric dating (^137^Cs or ^210^Pb) would allow on the more contaminated and shallower cores. Melosira (*M. granulata*) algae blooms in 1906 and 1982 in Lake Zurich were used as distinct event markers (Gammeter et al., 1997; Nipkow, 1927). The varve chronologies of ZH09-05 and ZH21-03 were checked against the varve chronology and Sn profile of core ZH10-15, a previously well-examined and dated core from Lake Zurich (Naeher et al., 2013) (Fig. 4). The stratigraphies of the three cores were not identical, because underwater mass movements have caused additional turbidite layers in ZH10-15, but less in ZH21-03 and none in ZH09-05 (Fig. 4) (Strupler et al., 2018; Naeher et al., 2013). Despite being further away from the silk dyeing factory, core ZH09-05 was finally used to date the contamination because its evolution was not interrupted by turbidites. The turbidite-rich core ZH21-03, however, was used to study the effects of mass movements on the contamination.

### Grain size distribution

Grain size distribution was measured with a laser diffraction particle size analyser (LS13 320 with Aqueous Liquids Module (ALM) and autosampler by Beckman Coulter Inc.). A Polarisation Intensity Differential Scattering detector (PIDS) was used for particles from 0.04 $\upmu$m to 0.4 $\upmu$m, and a light scattering detector (ISO 13320-1), for particles in the range 0.4 $\upmu$m to 2000 $\upmu$m.

Pea-sized samples of fresh sediment (no replicates) were suspended in ca. 5 mL 0.1% sodium polyphosphate solution and mixed overnight in an overhead shaker. The background (de-salted water) was measured for 60 s and compared to a factory reference background to ensure machine performance. The sample solution was then added to the sample chamber by autosampler, sonicated for 30 s, diluted to 50% PIDS obscuration, and measured for 90 s. The obtained light scattering pattern was then deconvoluted into a volumetric particle size distribution by the device software. Fractions of the volume distribution are grouped into the following categories: clay [0.04 ${\upmu \hbox {m}}$ to 2 ${\upmu \hbox {m}}$), silt [2 ${\upmu \hbox {m}}$ to 63 ${\upmu \hbox {m}}$), and sand [63 ${\upmu \hbox {m}}$ to 2000 ${\upmu \hbox {m}}$) (FAO, 2022).

### Bulk density and porosity

A Multi-Sensor Core Logger (MSCL) by GeoTek Ltd. with a gamma-source and detector was used to measure bulk density in unopened sediment cores (ZH08-16, ZH09-01–ZH09-06). The bulk-density module was calibrated using a sequence of aluminium cylinders with varying diameters. Density data was used to interpolate porosity measurements to whole sediment cores. The porosity was calculated using the weight difference before and after freeze-drying.

### Porewater measurement

Porewater was measured in two locations in Lake Zurich using dialyse plates (two plates for Sn and two plates for sulphate and sulphide measurement, respectively), so-called “peepers” (Urban et al., 1997). The peepers (Supporting Information Figure A4) used in this study were of the dimensions 15 cm × 64 cm × 2.5 cm. Each peeper has many small compartments that are separated from the sediment by a 0.2 ${\upmu \hbox {m}}$ polysulfone membrane (Gelman Inc.). The membrane allows for diffusion of dissolved substances and thus can be used to obtain a depth profile of dissolved substances in sediments. The plates were initially cleaned with HNO_3_, filled with Nanopure water and aerated with N_2_ for ca. 15 h to reflect the anoxic conditions in sediments and to avoid oxidation of dissolved species. The plates were installed by divers perpendicular to the sediment surface, partially sunk into the sediment, and fixed with buoys. The peepers were left to equilibrate for one week and then collected. 4 mL samples were collected per compartment and stabilised with 35 ${\upmu \hbox {L}}$ HNO_3_. Sn content was then measured using an Element 2 ICP-MS (Thermo Finnigan).

Sulphide was measured by colorimetry following a standard operating procedure based on Gilboa-Garber (1971). The 2 mL sample solution was stabilised with 0.5 mL zinc acetate solution and treated with a N,N-Dimethyl-1,4-phenylenediammonium dichloride salt solution to produce the reduced form of methylene blue, which was then oxidised by Fe(III). Sulphide content was determined photometrically using a U2000 Hitachi spectrophotometer by Boehringer Mannheim ($\lambda$ = 665 nm; limit of detection = 2.5 ${\upmu \hbox {mol}\, \hbox {L}^{-1}}$).

Sulphate was measured using an ion chromatography system (IC Metrohm Separation Center 733, Metrohm), equipped with a Metrosep Anion A supp. 5 column. The eluent was a bicarbonate/carbonate buffer. The water-sediment limit in the dialyse plates was determined by proxy using the concentration of phosphate and silicate. Both phosphate and silicate were measured by complexing with (NH_4_)_6_Mo_7_O_24_ and subsequent photometric measurement. Additionally, one core per location (ZH10-03 and ZH10-01) close to the peepers was retrieved using a gravity corer to compare Sn concentrations in the sediment with the adjacent porewater concentrations in the peepers.

Sn fluxes were computed to estimate the remobilisation of dissolved Sn from the sediment into the water column. Details of the calculation are provided in the SI.

## Results

### Spread of the contamination

The highest XRF Sn counts were found in front of the former silk factory (Fig. 2). The spread of the Sn contamination in the closer vicinity of the former silk dying factory can be mapped by the maximum XRF counts for Sn in the cores retrieved in the years 2019–2021 (Fig. 2A). Tin contaminated sediment layers in these cores showed a markedly dark red-brown hue. The lowest counts were observed in the northwest along the shore in core ZH19-31, and in the deep lake, in core ZH21-03. The Sn contamination was constrained to one distinct peak that started suddenly (ZH19-31: 10 cm, ZH19-35: 23 cm, ZH19-36: 33 cm) and disappeared slowly towards the top of the core (Figs. 3 and 4). Core ZH21-03, more disturbed by turbidites, displays several peaks of elevated Sn concentration: a first peak at 42 cm, and the most prominent peak at 33 cm in a turbidite layer. No correlated peaks could be found in the XRF profiles for other metals. The XRF Sn signals from earlier campaigns were not comparable in their value to the newer measurements due to changes in instrument setup. However, the XRF Sn profiles for most sediment cores reflected the ones seen in ZH19-36 (Supporting Information Figure A2). The downcore profiles of the Sn concentrations measured with ICP-OES in the samples of ZH19-31, ZH19-35 and ZH19-36 corresponded well with their respective XRF curves (Fig. 3). The maximum concentrations for Sn were 6.08 $\,{\hbox {g}\,\hbox {kg}^{-1}}$ (dry weight; for ZH19-31), 120 $\,{\hbox {g}\,\hbox {kg}^{-1}}$ (ZH19-35) and 177 $\,{\hbox {g}\,\hbox {kg}^{-1}}$ (ZH19-36).

Concentrations of other trace metals were lower than 800 ${\hbox {mg}\,\hbox {kg}^{-1}}$ in all measured sediment cores (Table 1), and do not show any correlation with the Sn profiles (see e.g. the downcore concentrations of Sn, Pb, Cr, Ni, Cu, and Zn in ZH19-35, Supporting Information Figure A5). Traces of the Sn contamination could be found throughout Lower Lake Zurich, even at the south end of the Lower Lake (0.05 ${\hbox {g} \,\hbox {kg}^{-1}}$ in ZH09-02), greatly exceeding natural levels in stream sediments in the region of Zurich, in the range 1 $\,\hbox {mg}\,\hbox {kg}^{-1}$ to 6 $\,\hbox {mg}\,\hbox {kg}^{-1}$ (Forum of European Geological Surveys (FOREGS) in Salminen et al. (2005)). No Sn XRF signals were detected in a core from Upper Lake Zurich (ZH09-01), which is why no further analyses were made on this core.

### Age of the contamination

The proximal sediment cores showed no clear laminations and little stratigraphic differences between each other (Fig. 3). However, distal sediment cores from the deepest part of Lake Zurich exhibit clear varves, and often turbidite layers as well (Fig. 4). Sediment cores ZH09-05 and ZH21–03 were thus used to establish a varve chronology. The average sedimentation rates in the varved sections obtained through varve counting were ca. 0.51 $\,\hbox {cm}\, \hbox {a}^{-1}$ and 0.28 $\,\hbox {cm}\, \hbox {a}^{-1}$, respectively, which is in line with previous varve chronologies from Lake Zurich (Naeher et al., 2013; Roethlin et al., 2022a).

In the lower part of the sediment cores ZH09–05, ZH10-15 and ZH21-03, the Sn concentration was at the XRF detection limit and most likely corresponded to the natural levels (Fig. 4). The onset of the Sn contamination could be clearly seen in core ZH09-05 and started 4 cm before the onset of varve deposition. To further constrain the date of the onset, we applied the sedimentation rate of the varved part (ca. 0.51 $\,\hbox {cm}\, \hbox {a}^{-1}$) to the pre-eutrophication part assuming a similar and constant sedimentation rate over these 4 cm. This suggest that the Sn concentration started rising in the early 1890s.

### Characterization of the Sn contamination

The comparison of TC, TIC, TOC, and TN contents in samples from the relatively uncontaminated site (ZH19-31) and in samples from the heavily contaminated site (ZH19-36) (Fig. 3) indicated that the TC content decreased downcore and TN remains relatively stable at both sites. However, the TOC content showed large variations only in the heavily contaminated core, increasing simultaneously with the Sn contamination.

The grain size distribution suggested that the Sn contamination is independent of grain size, since ZH19-31 and ZH19-36 exhibited similar clay, silt and sand fractions (Fig. 3). Both cores show a general trend towards less clay and more silt fractions towards the core top.

**Fig. 5:**
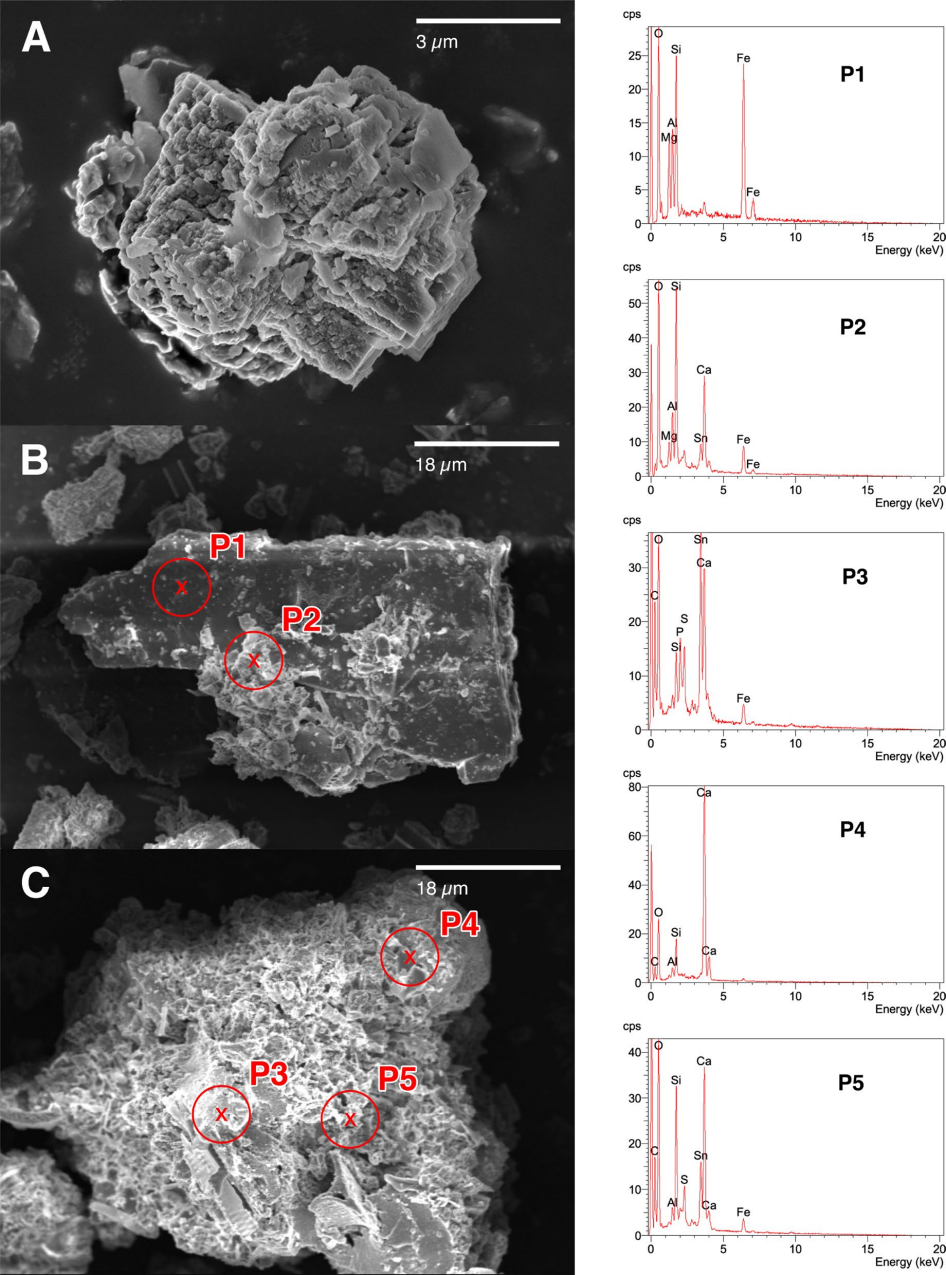
SEM images of contaminated sediment particles found in sediment core ZH05-57. **A** Calcite particle. **B** and **C** Aluminium silicate particles. The white particles are Sn. P1–P5 indicate points on the particles **B** & **C** that were used for further analysis with EDX. Corresponding EDX spectra of the surface of contaminated sediment particles at measurement points determined using SEM

The scanning electron microscopy measurements conducted on contaminated sediment samples from core ZH05-57 showed that aluminium silicates originating from soil particles run-off were covered with Sn particles (Fig. 5B, C). In contrast, biogenic calcite crystals were not coated with Sn particles (Fig. 5A). The two aluminium silicate particles in Fig. 5B and Fig. 5C were partially and fully covered with a Sn particle layer, respectively, as were most aluminium silicate particles found in the samples.

The five EDX measurements done on the sediment particles revealed EDX spectra typical for Ca-Al-Fe silicates in the case of P1 and P4, while the spectra for P3 and especially P5 revealed high Sn and S signals. In the case of P3, the Sn peak was the most prominent signal (Fig. 5).

**Fig. 6:**
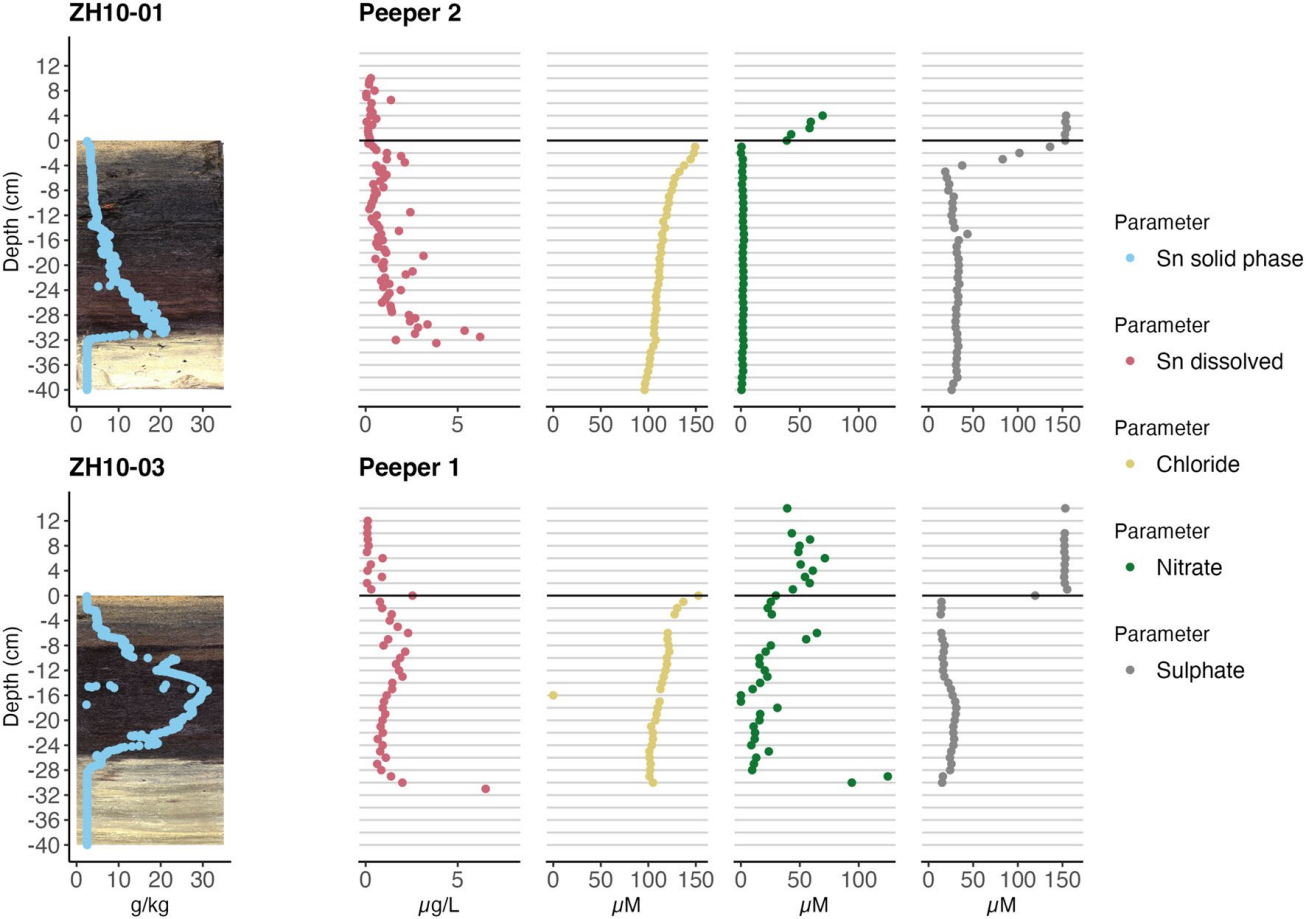
Porewater profiles for dissolved Sn, chloride, nitrate and sulphate for two locations. The total Sn concentration in the solid phase of the adjacent sediment cores is shown for comparison. The locations of peepers 1 and 2 are marked in Fig. 2C

Porewater measurements (Fig. 6) indicated that the soluble Sn fraction generally followed the total Sn contamination in the sediment cores, while the Sn concentration in the overlying water was very low (< 1 $\,{\upmu \hbox {g}\,\hbox {L}^{-1}}$). The Sn concentration peaked in the range of 20 ${\hbox{g}\,\hbox {kg}^{-1}}$ to 30 ${\hbox {g}\,\hbox {kg}^{-1}}$ at the two sites. At site 1, the Sn concentration in the solid phase increased below the surface to a depth of about 17 cm. By contrast, the Sn contamination at location 2 only started increasing at around 10 cm and peaked at 30 cm. For both locations, the soluble Sn concentration showed small sub-surface maxima and down-core concentration levels in the 1 $\,{\upmu \hbox {g}\,\hbox {L}^{-1}}$ to 2 $\,{\upmu \hbox {g}\,\hbox {L}^{-1}}$ range.

Porosities were estimated to 0.45 (Peeper 1) and 0.43 (Peeper 2) using MSCL measurements. The long-term diffusion fluxes were estimated to 0.06 ± 0.03 $\hbox {mg}\, \hbox {a}^{-1}\, \hbox {m}^{-2}$ (Peeper 1; diffusion zone 0 cm to 15 cm) and 0.04 ± 0.02 $\hbox {mg}\, \hbox {a}^{-1}\, \hbox {m}^{-2}$ (Peeper 2; diffusion zone 0 cm to 31.5 cm). The sulphate and chloride profiles showed a clear decrease in sulphate and chloride at the water-sediment interface, which are due to today’s higher Cl^-^ loads compared to the past, and sulphate reduction in the anoxic sediments. No sulphide could be measured in either location (conc. < 2.5 ${\upmu \hbox {mol} \, \hbox {L}^{-1}}$), which is not surprising in such metal-rich sediments.

## Discussion

### Source of the contamination

The distribution pattern with the highest concentrations in front of the former silk factory in Thalwil pointed to this factory as the source of the widespread Sn contamination in Lake Zurich. However, textile factories and chemical industries were ubiquitous at the dawn of industrialisation in Switzerland (ca. 1850–1900, cf. Introduction). Several polluting factories were located around Lake Zurich during and after the Industrial Revolution, for instance a paper factory in Horgen, a chemical plant in Uetikon am See, or a cotton printing factory in Richterswil, which was later replaced by a rubber factory (see locations in Fig. 1B). As such, it is currently unclear if other factories contributed to the Sn contamination in Lake Zurich, since Sn was found both upstream (southeast) and downstream (northwest) of the silk factory. The wide distribution indicates that the Sn was not dumped in soil material (e.g. during land backfilling) as observed by Roethlin et al. (2022a) near the shores of Richterswil, but released as a solution or suspension into the water. The downcore TOC distribution at the heavily contaminated site suggested simultaneous sedimentation of organic material (e.g. from tannins used for silk weighting) with the Sn contamination.

While Sn could be found everywhere in Upper Lake Zurich, the large difference in Sn concentrations in adjacent sediment cores (Fig. 2 and Table 1) and its rapid decline from the shore away suggest that the original Sn compounds were poorly soluble, or rapidly transformed and sedimented. A quick Sn sedimentation is supported by the scanning electron microscopy measurements that have been conducted on contaminated sediment samples of core ZH05-57. The presence of Sn particles on the aluminium silicates (Fig. 5) and their absence on biogenic calcite suggested that the Sn quickly precipitated on geogenic material.

### History of the contamination

According to our extrapolated varve chronology, the Sn contamination started in the early 1890s. At that time, the tin-tannin weighting and the very popular, newly patented tin-silicate-phosphate weighting method (1893) were most likely used in the rapidly-growing silk dyeing factory in Thalwil. The Sn levels in the sediments then quickly increased, and the maximal Sn concentration was found around 1900, corresponding well with the golden age of the *Färberei Weidmann*. After that, the Sn concentration started gradually decreasing, going back to low levels in the 1940s. The reduction of the pollution intensity most likely reflected the slow decay of the silk industry. Silk deterioration caused by tin weighting was clearly identified in 1905, which harmed the reputation of the silk industry (Meister, 1905). In addition, the reduced use of tin weighting, the recuperation of Sn from used baths, and the development of artificial silk were reflected in the gradual decrease of the Sn concentration in the sediments. The First World War, the Great Depression and the Second World War also led to decreases of industrial activities.

The contamination history in cores ZH10-15 and ZH21-03 was comparable to the one in ZH09-05 (Fig. 4). However, several Sn peaks, including the ones with the maximal Sn concentrations, were found in turbidite layers. The Sn peaks seen in the turbidites of the sediment cores ZH21-03 and ZH10-15 (Fig. 4) indicate that the spreading of the Sn contamination from the shore in Thalwil toward deep water happened not only via sedimentation, but also via mass movements. For instance, mass movements from 1909 and 1913 remobilised highly-concentrated sediments from the shore of Thalwil and redeposited them as turbidites in the deep lake, as seen in ZH21-03, with Sn concentration higher than the underlying sediment. These mass movements were promoted by modifications of the lake shore, such as embankments to make room for roads and railways. The *Färberei Weidmann* itself also reclaimed land from the lake by infilling to extend the factory. With the decrease in new embankments over time, associated mass movements became rarer. In the absence of mass movements, the turbidite-free sediment core ZH09-05 indicates that the contamination rapidly increased and then slowly decayed. So, the high Sn peaks in the turbidite layers of ZH21-03 and ZH10-15 do not, in fact, reflect maximum Sn concentrations, but rather represent the Sn physically remobilised by mass movements.

**Table 1:** Maximum concentration of different trace metals measured with ICP-OES and ICP-MS in sediment cores of Lake Zurich. All concentrations are given in ${\hbox {mg}\,\hbox {kg}^{-1}}$

Core	Cd	Sn	Pb	Cr	Ni	Cu	Zn
ZH08-16	1.48	2550	55.7	43.8	25.1	35.9	112
ZH09-02	0.404	493	77.5	32.8	13.4	24.7	102
ZH09-03	0.508	1050	102	61.5	25.8	49.6	102
ZH09-05	2.57	1220	81.2	56.4	23.4	46.4	144
ZH09-06	0.457	875	93.1	41.7	18.5	52.6	78.9
ZH10-01	0.728	30 500	332	53.2	20.1	210	249
ZH10-03	0.811	29 700	739	62.6	15.2	691	337
ZH10-05	1.24	15 400	255	53.9	18.2	186	209
ZH10-10	0.771	11 700	116	41.5	24.1	74.8	157
ZH10-11	0.787	562	96.4	36.5	21.0	37.8	203
ZH19-31	5.04	6080	169	104	35.8	65.7	292
ZH19-35		118 000	500			271	321
ZH19-36	5.32	177 000	730	106	40.2	479	390

### Risk assessment

Elements of a risk assessment of the Sn contamination include a worst-case estimate of the expected sedimentary flux, the potential toxicity of an estimated Sn concentration in the lake water, the long-term fate of the Sn deposits, and the risk of potential Sn methylation. An estimated average diffusion rate from the two peeper measurements corresponded to 0.05 $\hbox {mg}\, \hbox {a}^{-1 } \,\hbox {m}^{-2}$. When scaling this flux to the surface area of the whole lake (68 ${\hbox {km}^{2}}$), we obtained a worst-case scenario of 3.4 kg Sn mobilised per year, which would lead to an increase of the Sn concentration of only 1 ${\hbox {ng}\, \hbox {L}^{-1} \,\hbox {a}^{-1}}$ in the fully mixed lake (3.4 ${\hbox {km}^3}$), or 1.4 ${\hbox {ng}\, \hbox {L}^{-1}}$ over the water residence time of 1.4 years. The local concentration at the contaminated sites 1 and 2 was below 1 ${\upmu \hbox {g}\, \hbox {L}^{-1}}$ in the porewater at the sediment-water interface, which would be rapidly diluted by turbulent mixing in the overlying water. Sn(IV) species would be present under oxic conditions, but their toxicity to aquatic life is very low (Rüdel, 2003) with $EC_{50}$ values of 22 ${\hbox {mg}\, \hbox {L}^{-1}}$ for daphnids. In the case of human toxicity, experience in food packaging indicates a low health risk with permissible Sn levels of 150 ${\hbox {mg}\, \hbox {L}^{-1}}$ for beverages (Blunden and Wallace, 2003).

The high total Sn concentration in contaminated sediments, however, required an assessment of its long-term fate and bioavailability to sediment-dwelling organisms. The SEM-EDX measurements revealed a high sulphur content of the Sn phases. An analysis of the Sn-S-O-H system ($C_{\text{S}}$ = $10\times 10^{-3}\,{\hbox {mmol}\, {\text{L}}^{-1}}$, $C_{\text{Sn}}$ = $10\times 10^{-8}\,{\hbox {mmol}\, {\text{L}}^{-1}}$; Supporting Information Figure A6.B) showed that the Sn is likely bound as SnS_2_ in the sediment under anoxic conditions and in presence of sulphide. With a solubility product of $pK_s = 70$, Sn(IV) is expected to be effectively trapped in this disulphide phase (Blais et al., 2008), limiting the dissolution and bioavailability of Sn in the contaminated layers. In addition, the Sn layers have been covered with uncontaminated sediment over the past decades.

Unlike inorganic Sn, organotins are toxic to various degrees, and some can be produced through methylation under anoxic conditions by sulfate-reducing bacteria (Gilmour et al., 1987). If we assume that the entirety of the Sn remobilised in porewater is methylated—which is however very unlikely, we obtain values of 1.4 ${\hbox {ng}\,\hbox {L}^{-1}}$, much below the recommended value of 0.6 ${\upmu \hbox {g}\,\hbox {L}^{-1}}$ as Sn for the total organotin content in drinking water (World Health Organization, 2020). In their experiments, Gilmour et al. (1987) found that only 0.02% of the inorganic Sn added to the sediment slurries was methylated and resulted predominantly in monomethyltin, while the more toxic di- and trimethyltin were produced more sparingly. In the highly contaminated sediments near Thalwil, this could result in organotin levels of $\sim$20 ${\upmu \hbox {g}\,\hbox {g}^{-1}}$ as Sn. However, ecotoxicological experiments on those sediments showed no increased ostracod mortality in the contaminated layers (Roethlin, 2023). In addition, because of the low and stable levels of highly substituted methyltins, Ashby and Craig (1988) also expect the existence of demethylation processes in the environment.

In summary, we observe low Sn remobilisation in porewater, limited bioavailability of Sn in the contaminated layers, and low risks linked to methylation. Overlying uncontaminated layers are likely to assure a safe burial of this industrial legacy over the next centuries, provided that the contaminated layers are not perturbed, notably by mass movements. Therefore, we conclude that the Sn contamination, caused a century ago by the silk dyeing industry on the shore of Lake Zurich, currently poses no threat to the aquatic ecosystem nor to the production of drinking water.

## Conclusions

In summary, we could show that the Sn contamination in the sediments of Lake Zurich is present in the entire Lower Lake but is increasing toward the shore of Thalwil. The contamination is constrained to one peak: the Sn concentration started rapidly increasing around 1893, reached a maximum around 1900, and then decreased slowly until it returned close to background levels in the 1940s. We conclude that the main cause of the Sn contamination is the silk dyeing industry *Färberei Weidmann* in Thalwil, which used significant Sn amounts for silk weighting from 1893 on, before declining in the 20^th^ century. We observed that the Sn in contaminated sediment layers can be physically remobilised by mass movements, as indicated by the higher Sn concentration in turbidites. It is therefore crucial to prevent new mass movements, in particular in the vicinity of Thalwil. They are likely the main risk of Sn remobilisation, because the contaminated layers have since been covered with uncontaminated sediments and the Sn concentration in porewater is low. Combined with the low toxicity of inorganic Sn, these observations allow us to conclude that the Sn contamination is neither of concern for the lake ecosystem nor for drinking water production, unless major mixing processes bring sediments back to the oxic zone.

## Supplementary Information

Additional file 1.

## Data Availability

The data that supports this study has been deposited on the Eawag Research Data Institutional Repository, which is a FAIR Open Research Data repository. The doi associated with this dataset is: 10.25678/000DAK. Supporting information: the following file is available free of charge. CoreOverview.pdf: Overview of sediment cores (linescans) used in this study, including XRF Sn profiles for ZH08-16, ZH09-XX, ZH19-XX and ZH20-XX cores, and XRF profiles for core ZH19-35 for Sn, Pb, Cr, Ni, Cu, and Zn (Appendix A). CoreOverview.pdf: Table containing depths and coordinates for all sediment cores used in this study (Appendix B).
