# Supplementary material for: Tin contamination in sediments of Lake Zurich: source, spread, history and risk assessment
Source: Swiss J Geosci. 2024 Dec 24;117(1):22. doi: 10.1186/s00015-024-00471-6 (PMC11668850; doi:10.1186/s00015-024-00471-6)
Supplement: Supplementary file 1 — Additional file 1. [file 15_2024_471_MOESM1_ESM.pdf]

Supporting Information for: Tin contamination in sediments of Lake Zurich: source, spread, history and risk assessment

Remo Roethlin, Aurélia Meister, Adrian Gilli, Sinikka Lennartz, Maria Dittrich, Eri Amsler, Bernhard Wehrli, Maria Schönbächler, Nathalie Dubois

A Figures

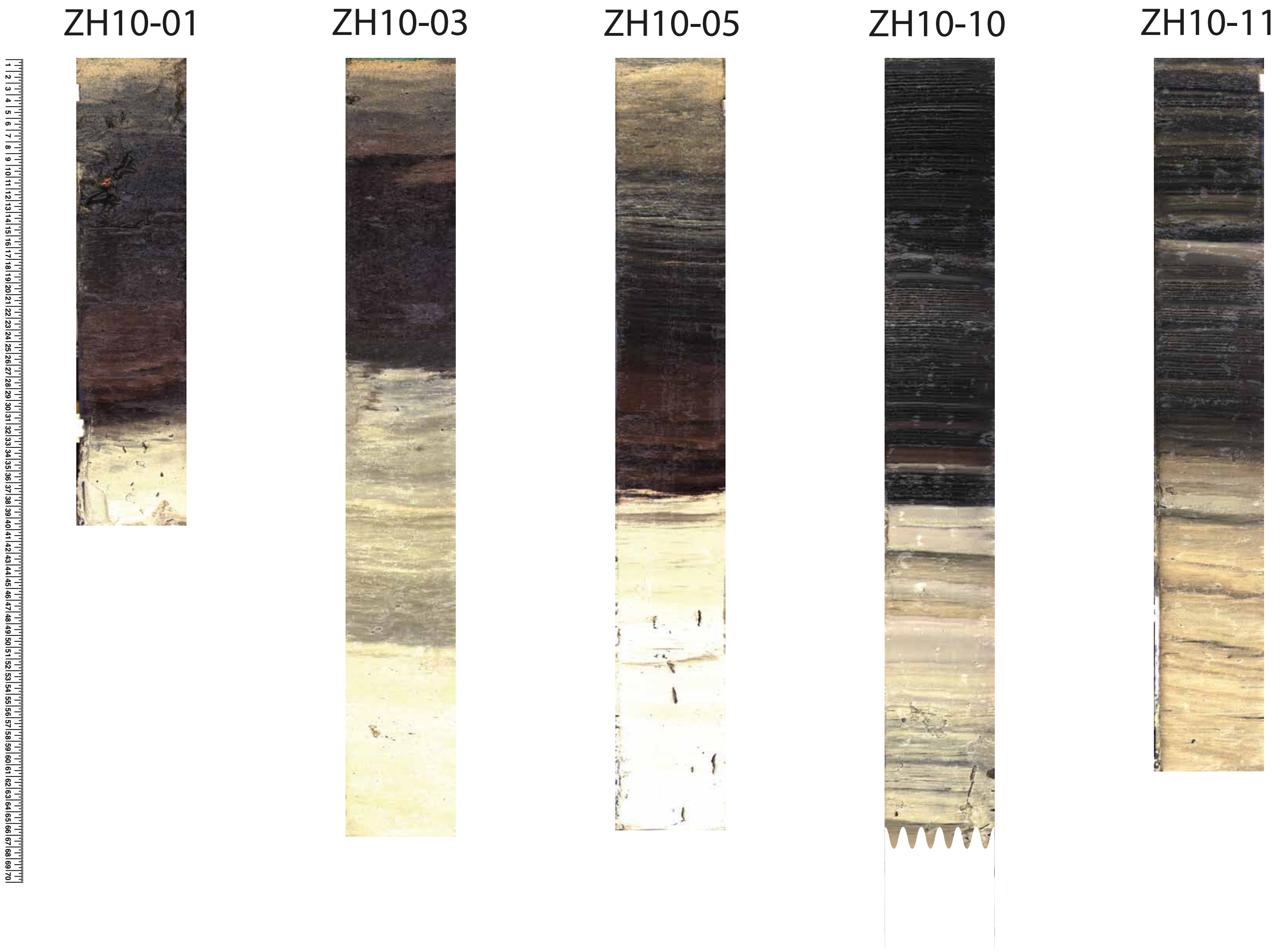

Figure A1: Overview of sediment cores from around Thalwil and Lake Zurich.

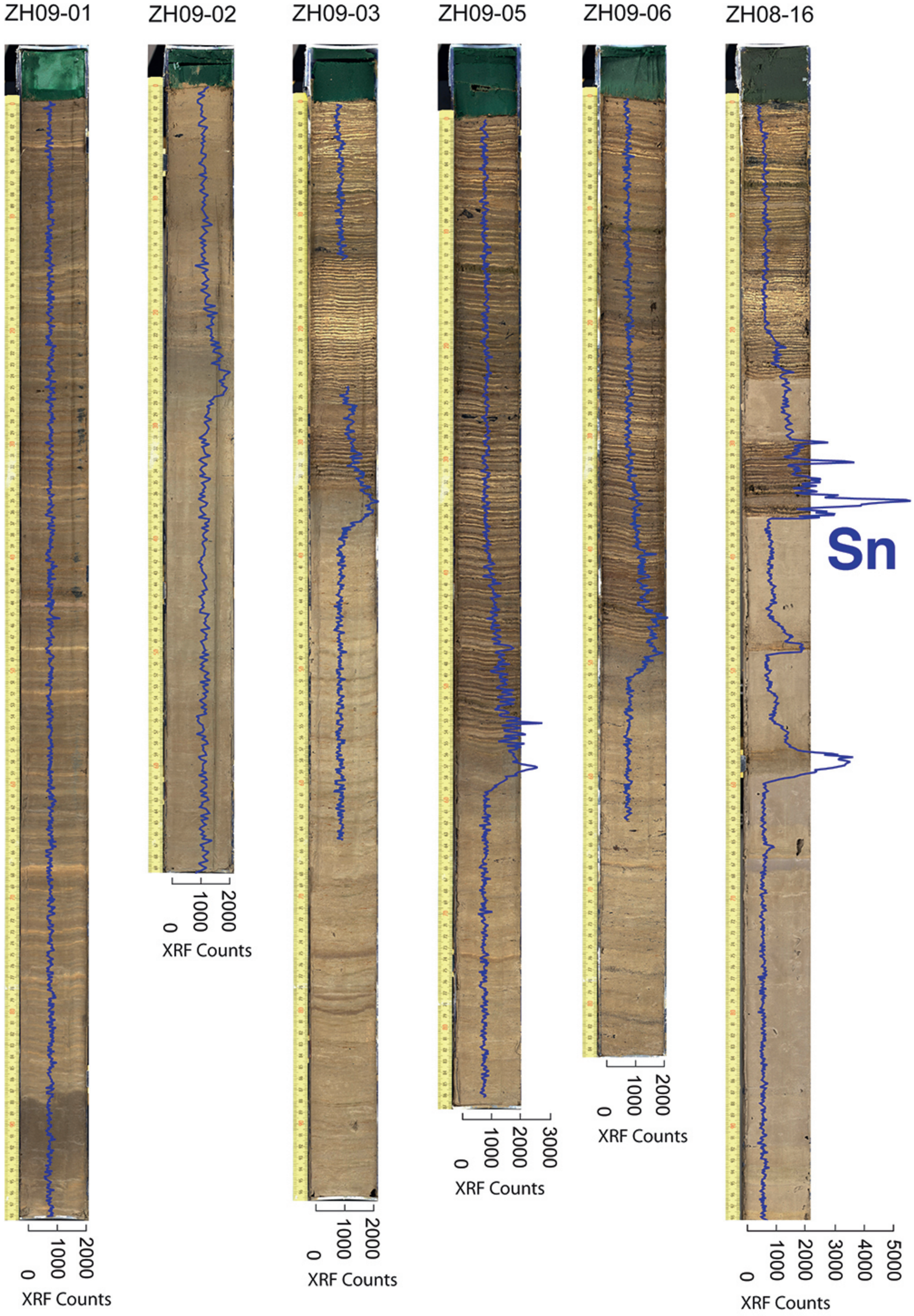

Figure A2: Overview of Lake Zurich sediment cores taken in 2008 and 2009 with their respective XRF Sn profiles.

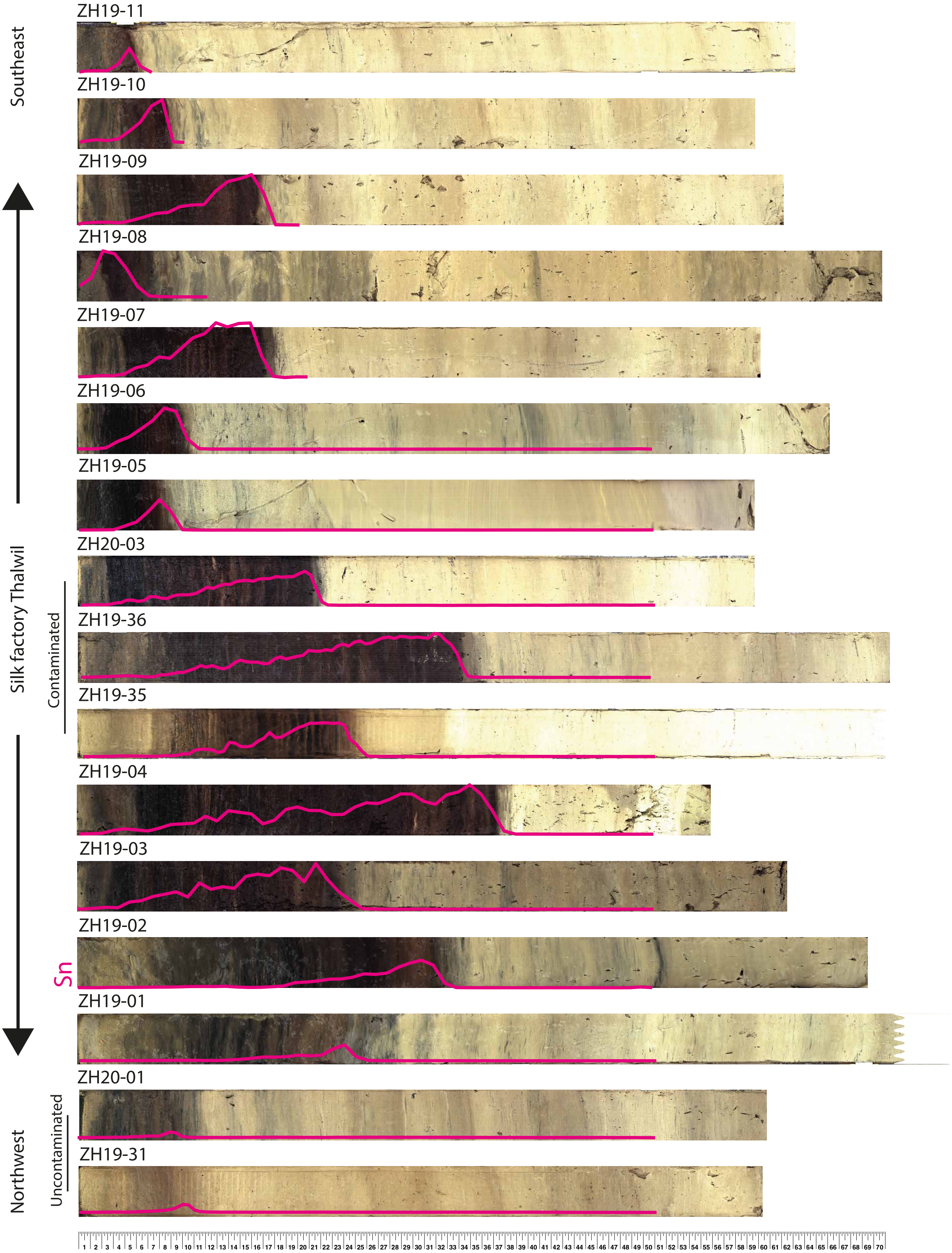

Figure A3: Overview of Thalwil sediment cores taken in 2019 and 2020 with their respective XRF Sn profiles.

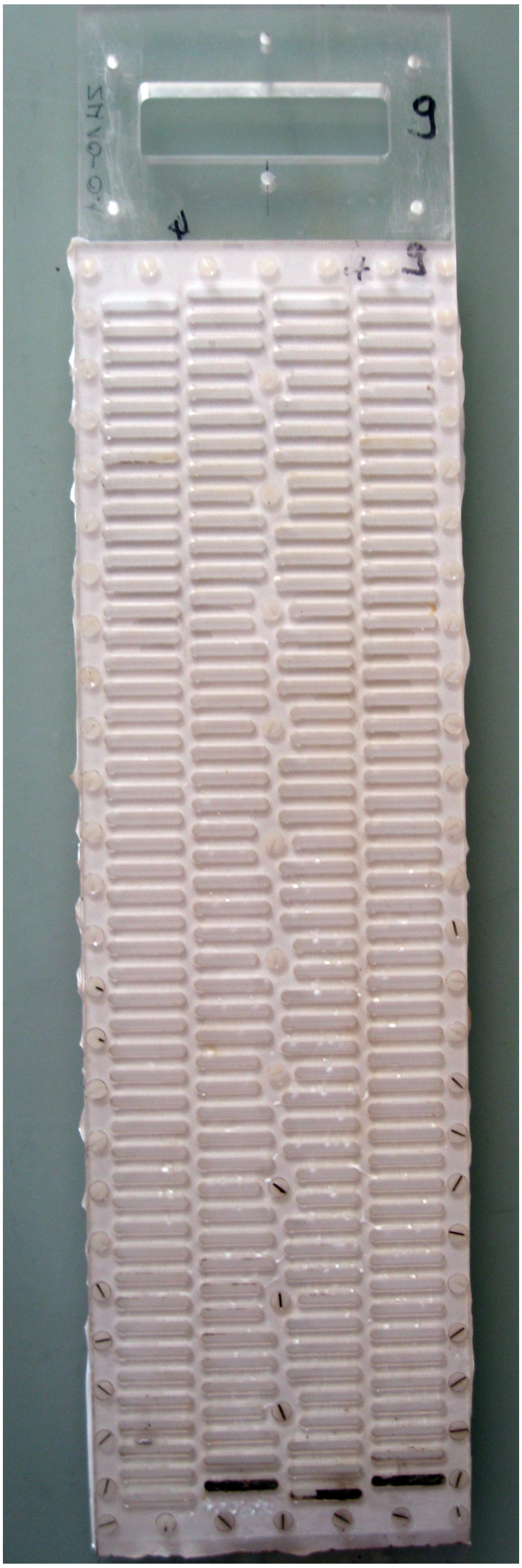

Figure A4: Photograph of a "Peeper"/dialyse plate used for porewater measurements.

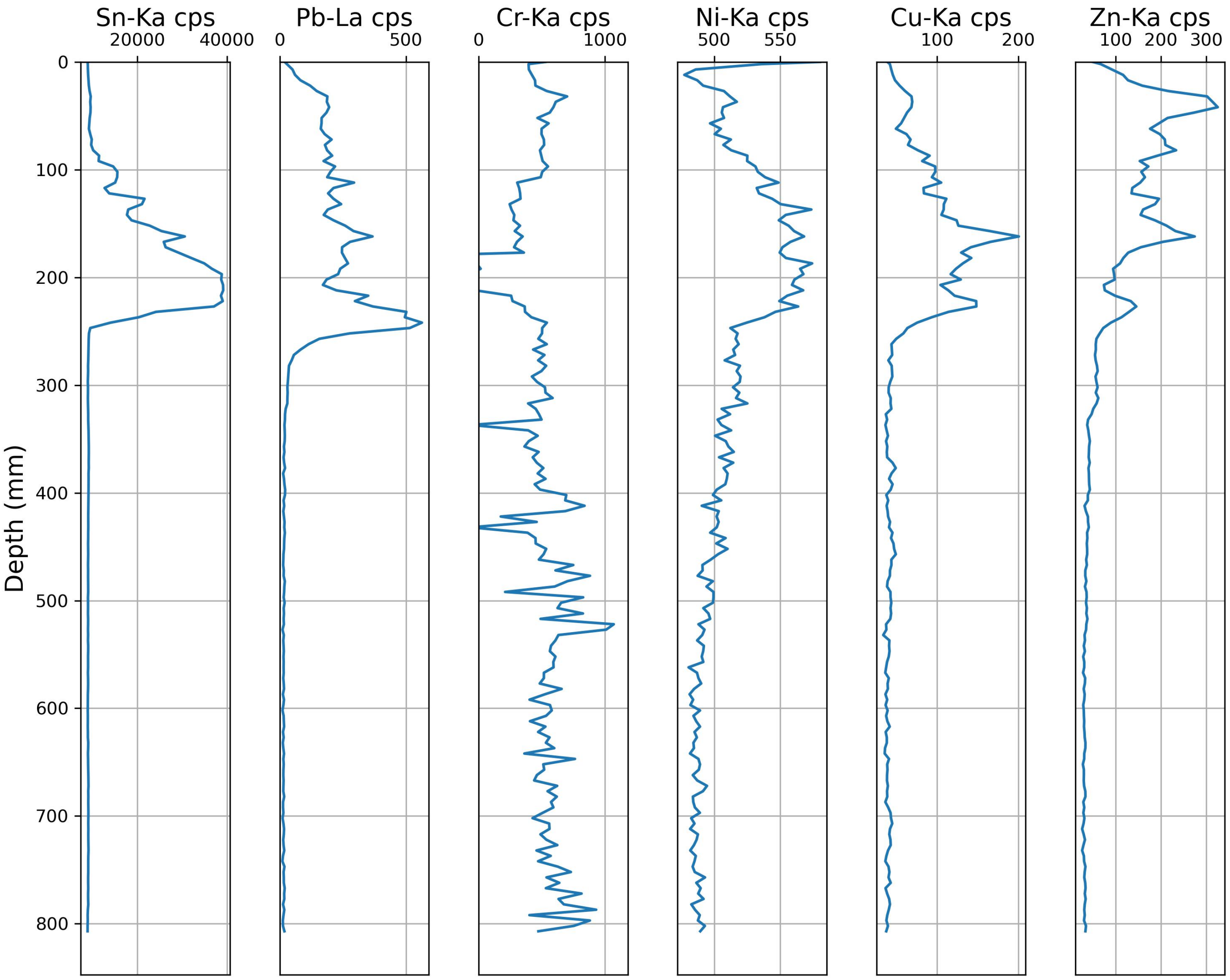

Figure A5: XRF profiles for Sn, Pb, Cr, Ni, Cu, and Zn in core ZH19-35. The XRF profiles for Sn, Pb, Cr, Ni, Cu, and Zn in core ZH19-35 show that the Sn signal is orders of magnitude higher than those of the other metals, emphasizing the extent of Sn contamination. No other metal fully correlates with the Sn profile. Pb exhibits the most similar trend to Sn; however, significant differences are observed. Pb starts to increase slightly deeper in the core and shows a sharp decrease at the depth where the Sn concentration peaks. Furthermore, Pb remains relatively constant with little to no decrease towards the top of the core, while Sn declines rapidly and steadily after the initial peak and approaches background levels within the upper 80 mm. In contrast, Cr shows no discernible pattern and exhibits high variability, resembling a sawtooth profile. Ni and Cu share some similarities with each other, with their peaks located around 200-100 mm depth. Zn, on the other hand, increases primarily in the upper 100 mm of the core, which distinguishes it from the other elements. While we present these profiles for additional context, it is important to note that a detailed discussion of these elements and their variations falls outside the scope of this manuscript, which focuses on the Sn contamination.

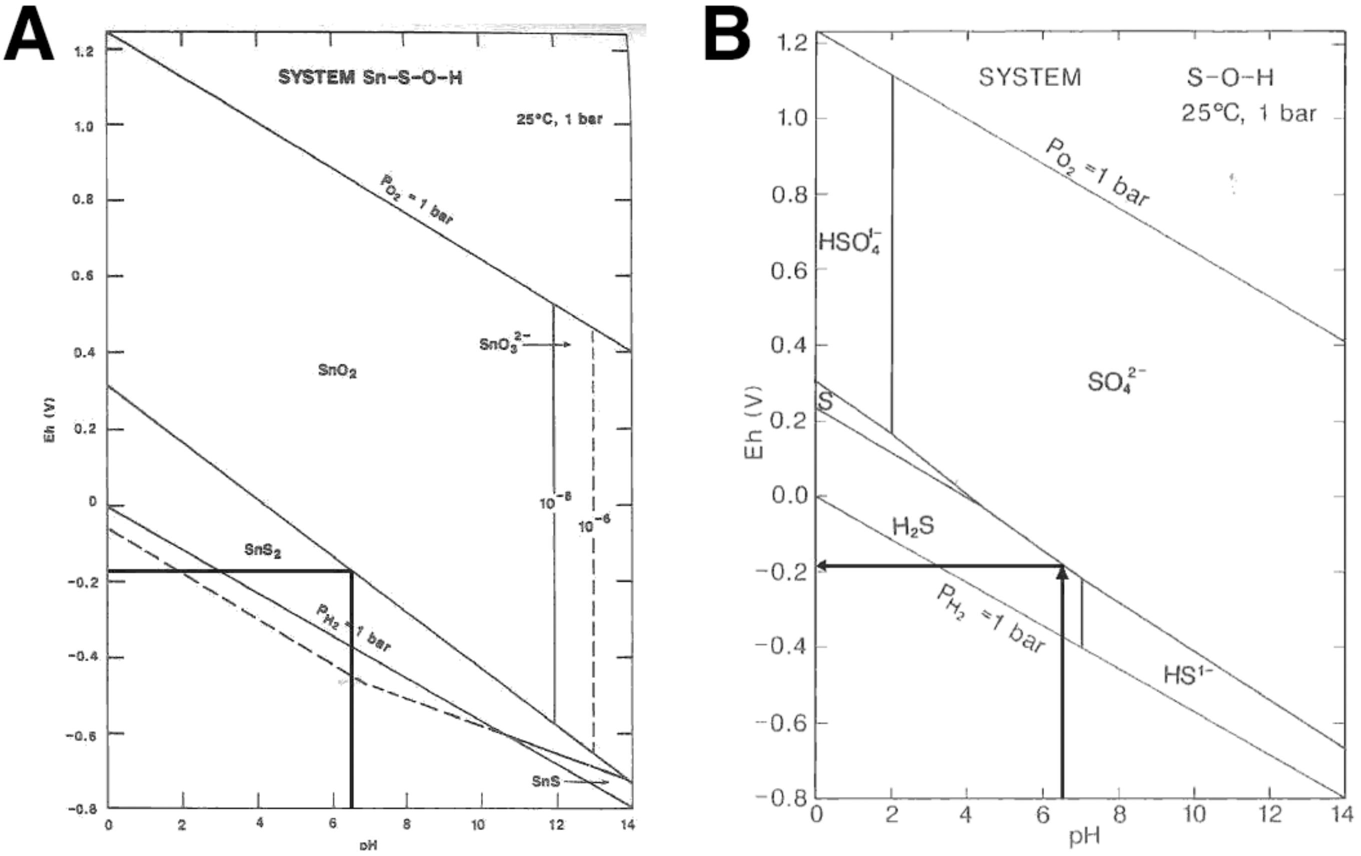

Figure A6: Pourbaix diagrams for the systems S-O-H (subfigure A) and Sn-S-O-H (subfigure B), modified after Brookins (2012). Concentrations: $C_S = 10 \times 10^{-3}\,\mathrm{mmol\,L^{-1}}$, $C_{Sn} = 10 \times 10^{-8}\,\mathrm{mmol\,L^{-1}}$.

B Tables

Table B1: Overview of sediment cores with their respective water depth and coordinates.

Latitude	Longitude	CoreID	Depth
47.21423	8.90924	ZH09-01	49.1
47.22154	8.72831	ZH09-02	24.8
47.24075	8.67869	ZH09-03	65.2
47.25879	8.64451	ZH09-04	124
47.28542	8.60226	ZH09-05	126.6
47.28536	8.60349	ZH09-06	118
47.28494	8.59450	ZH08-16	139
47.29295	8.57321	ZH10-01	30.5
47.29293	8.57246	ZH10-03	17.5
47.29333	8.57394	ZH10-05	35
47.29725	8.58234	ZH10-10	35.5
47.31662	8.56787	ZH10-11	31.5
47.30222	8.56365	ZH19-31	8.5
47.29474	8.57185	ZH19-36	18.5
47.29532	8.57252	ZH19-35	26.5
47.29451	8.57214	ZH05-57	
47.28383	8.59379	ZH10-15	137
47.29308	8.57246	Peeper 1	11.2
47.29295	8.57321	Peeper 2	18.3
47.29656	8.58043	ZH21-03	132
